# Co expression of EGFR and CD10 in patients with phyllodes tumors of the breast: a single center experience in North Western Algeria

**DOI:** 10.4314/ahs.v23i4.29

**Published:** 2023-12

**Authors:** Amina Belhadj, Samia Bekkal Brikci, Chahinez Akhrouf, Adel Belhadj, Miloud Medjamia, Tewfik Sahraoui

**Affiliations:** 1 Biology of Development and Differentiation Laboratory, Oran 1 University, Ahmed Ben Bella, Department of Biology, Faculty of Nature and Life sciences; 2 Faculty of Medicine, University of Oran 1, Ahmed Ben Bella Algeria; 3 Anatomy pathology laboratory. Military hospital. Oran. Algeria

**Keywords:** Breast phyllodes tumors, cluster of differenciation10, epidermal growth factor receptor

## Abstract

**Background:**

Breast phyllodes tumors (BPT) have variable malignant potential, their histological classification remains insufficient for an accurate diagnosis.

**Objectives:**

We attempted to investigate CD10 (Cluster of differentiation 10) and EGFR (Epidermal growth factor receptor) expression in BPT in order to highlight their diagnostic and prognostic values.

**Methods:**

Eight patients with BPT are recruited from January 2014 to December 2020 and immunohistochemical assessment of CD10 and EGFR is realized.

**Results:**

Median age was 27±15.2, the mean tumor size was 9.63±10.21. Only malignant tumours showed expression for EGFR. Borderline and malignant tumors were CD10 positive. Patients overexpressing CD10 were postmenopausal with great tumor size, 25% of these were sarcomatous. Coexistence of CD10 and EGFR overexpression was found in 25% of cases and was associated with age (P=0.008), tumor size (P=0.030) and hitologic types (P=0.014). PC1 and PC2, were extracted, they accounted cumulatively for 94.7% of the variance of the data analysed, it suggests that patient's age and histological type of tumor have significant association with CD10 and EGFR expression in BPT.

**Conclusions:**

EGFR and CD10 overexpressed combined proteins in phyllode tumors constitute, with histopathological parameters, an important prognostic factor as well as a promising potential targets.

## Introduction

Breast cancer is diagnosed at late stages in Algeria, its incidence is about 21.5%^1^. BPT are extremely rare biphasic tumours that represent 0.2% to 2 % of all breast tumours. Their pathogenesis and underlying genetic alterations were misunderstood^2^. BPTs are classified as benign, borderline or malignant according to histologic features^3^. The tumor grade, i.e., benign, borderline or malignant, is based on histologic features including cellularity and atypia of the stroma, tumor border, malignant heterologous elements and mitotic activity^4^. The differentiation of benign BPT, borderlines and malignant in pre-operative is a major interest in surgical indications of tumorectomy, for the choice of margins of removal and additional treatment indications to reduce the risk of recidivism^5^. Borderline and malignant BPTs remain to this day difficult to classify, standard histological interpretation may vary when based on morphological criteria alone, particularly among relatively inexperienced pathologists, therefore, this can induce to real problem of diagnosis and to a bad therapeutic management. Several biological markers have been explored to distinguish between different grades of BPT, this, contributes to decrease the risk of underestimating the grade of phyllodes tumours and helps oncologists to choose the more appropriate treatment. Among these markers, EGFR, which belongs to the receptor tyrosine kinase family^6^, is involved in the pathogenesis and progression of mammalian malignant tumours^7^, for phyllodes tumours of the breast, the expression of EGFR appears to be amplified according to tumor grade to be maximum in malignant tumors^8^,^7^. CD10 is a matrix metalloproteinase that cleaves the protein components of extracellular matrix and thereby plays a central role in tissue remodeling^9^. Stromal CD10 expression is also associated with biological aggressiveness in various epithelial malignancies including the breast as it is overexpressed in malignant, compared to borderline and benign tumors^10^. Few studies have highlighted the role of biomarkers in the categorization of BPTs in addition to the fact that some of the studies have controversial results, in our current study, we aimed to evaluate for the first time in Algeria, the prognostic values of EGFR and CD10 among Algerian patients in correlation with the tumor size, histological type and patient's age of the mammary phyllodes tumors.

## Methods

In the current study we performed immunohistochemical staining of EGFR and CD10 in eight BPTs diagnosed between January 2014 and December 2020 at department of pathology of Oran Military Hospital (Western Algeria). All tumor specimens were fixed in buffered formalin and embedded in paraffin. All cases were reviewed and all the diagnoses were reconfirmed. An absolute confidentiality of the patients' vital information was maintained for ethical purposes and an ethical approval was obtained from the institution in which the study was carried out.

### Immunohistochemical staining and evaluation

The formal in fixed, paraffin embedded tissue sections (3 Am thick) were deparaffinized and hydrated, washed once in H2O for 5 minutes and then incubated in target retrieval solution Citrate (pH 6.0) in a boiling water bath for 50 minutes. The slides were cooled for 20 minutes. Following washing in phosphate buffered saline (PBS), slides were incubated with 3% hydrogen peroxide in methanol for 10 min to block endogenous peroxidase activity and washed again in H2O for 5 minutes and then incubated with a blocking serum for 10 minutes. Anti CD10(ready to use, Clone 56C6, Dako) and anti EGFR (1 :100 dilution, Clone HM, Dako) mouse monoclonal antibodies were applied, then incubated for one hour and washed in PBS for 5 minutes, then incubated in secondary antibody (Dako REAL™ EnVision™ Detection System) at room temperature for 30 minutes. The slides were washed and developed in 3,30 diaminobenzidine (Peroxidase DAB, Rabbit Mouse purchased from Dako.) under microscopic observation. The reaction was stopped in tap water and the tissues were counterstained with Mayer's hematoxylin, dehydrated, and mounted. The images were taken using Leica DFC 280 (Leica DM LB2). The expression was quantified blindly by two observers. Immunostaining of CD 10 was evaluated in cytoplasmic component whereas that of EGFR in membranous and cytoplasmic ones with a positivity threshold of 10% for both.

### Statistical analyses

The χ^2^ test was used to investigate the significance of the relationships between biomarkers expression and individual variables. Principal components analysis (PCA, for short) was used to identify the main axes of variance within a data set and allows for easy data exploration to understand the key variables in the data and spot outliers. Statistical analyses were done using SPSS 20.0 (Statistical Package for the Social Sciences, IBM Corporation; Chicago, IL. August 2011) and a probability of p < 0.05 was regarded as significant.

## Results

### Demographic characteristics

**Figure 1:**
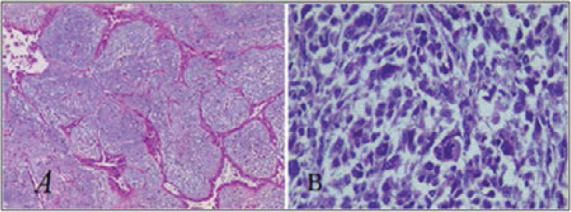
Representative histologic images of Hematoxylin and eosine staining of phyllodes tumors of the breast. A) Benign phyllode tumor with predominance of conjunctive tissue with discrete atypia and low mitotic activity (X100), B) phyllode sarcoma. Mesenchymal undifferentiated tumor with marked cytonuclear atypia (X400)

Patients were aged from 25 to 60 years old, median age was 27±15.29. Microscopically, 5 tumors were benign (Figure 1a), one was borderline and two tumors were malignant (Figure 1b). Malignant tumors showed marked cellular pleomorphism, had rich vascularization with infiltrating intercrossed fascicles. One of the two patients with the sarcoma-like phyllode tumour represented Paget disease with infiltrating axillary lymph nodes and a high proliferation marker index (Ki 67=50%).

### Immunohistochemistry expression of CD 10 and EGFR

In the entire series of the study, EGFR and CD10 expressions were noted in 2 cases (25%) and 3 cases (37.5%) of BPTs, respectively.

### EGFR expression

**Figure 2:**
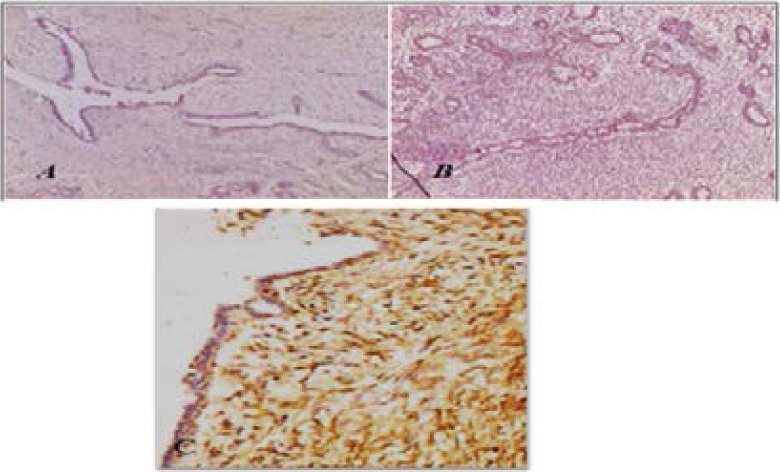
EGFR immunostaining in Breast phyllodes tumors. A) Negative in benign phyllode tumor (X100). B) Negative in borderline phyllode tumor (X100). C) Positive in malignant phyllode tumor(X200)

Our immunohistochemical analysis revealed that EGFR protein doesn't show any immunostaining intensity in beginning and borderline BPTs, however we have noted a strong EGFR expression in sarcomatous phyllodes tumors (Figure 2). There was a significant correlation between EGFR expression and age of patients (P=0.035), histological types (P=0.018) but not with tumor size (P=0.108) (Table 1). We have also noted that tumors overexpressing EGFR had a high mitotic index (data is not shown).

**Table 1:** Association of EGFR expression and clinicopathological features

Pathological parameters		EGFR − n (%)	EGFR + n (%)	P-value
Age	<50	5 (83.3%)	0 (0%)	0.035
	≥50	1 (16.7%)	2(100%)	
Histological type	Benign	5 (83.3%)	0 (0%)	0.018
	Borderline	1(16.7%)	0 (0%)	
	Sarcoma	0 (0%)	2 (100%)	
Tumor size	≤2 cm	2(33.3%)	0 (0%)	0.108
	2-5 cm	3(50%)	0 (0%)	
	≥5 cm	1(16.7%)	2 (100%)	

### CD10 expression

All benign phyllodes tumours did not express CD10 in their cytoplasm, except one case which showed positive myoepithelial immunostaining.

**Figure 3:**
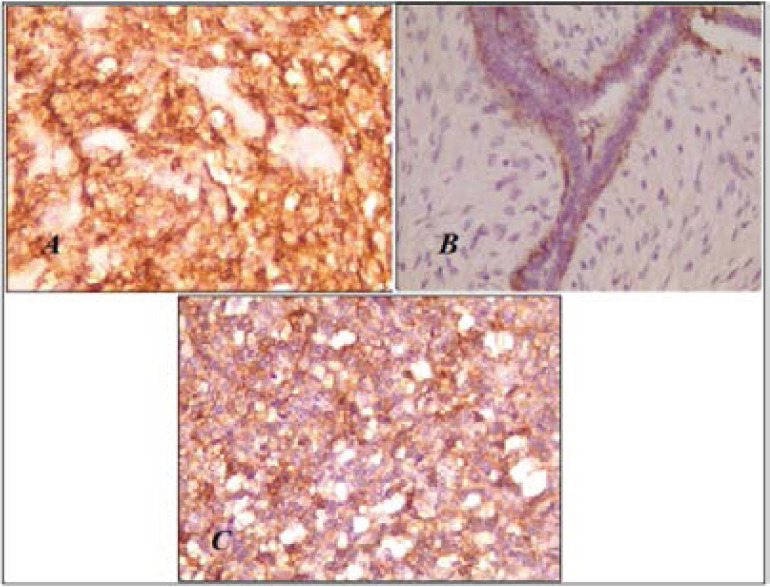
CD10 protein expression in Breast phyllodes tumors. A) Borderline phyllode tumour: Immunopositivity of CD 10 on the basal epithelial pole and myoepithelial cells X100. B) Negative immunostaining in benign phyllode tumor X400. C) Strong cytoplasmic CD10 immunostaining in malignant phyllode tumor X400

For borderline tumours, all showed mesenchymal CD10 immunopositively staining with low intensity in the myoepithelial component, CD10 was strongly expressed in the cytoplasm of sarcoma phyllodes (Fig 3). A significant correlation was found (P=0.0138) when CD 10 immunostaining status was compared between the three phyllodes tumors (Table 2) and also between benign vs borderline/malignant phyllodes tumors (P=0.005) (Table 3).

**Table 2:** Immunohistochemistry of CD10 expression in benign, borderline and malignant Phyllodes tumors

		BPTs (number)			P-value
		Benign	Borderline	Sarcoma	
**CD10**	-	5	0	0	0.0183
	+	0	1	2	

**Table 3:** Comparison of CD10 immunohistochemistry expression in borderline and malignant phyllodes tumors with benign phyllodes tumors

		BPTs (number)		P-value
		Benign	Borderline/ Malignant	
**CD10**	-	5	0	0.005
	+	0	3	

There were statistically significant differences between CD10 expression and pathological features, patients with tumors overexpressing CD10 were all postmenopausal (P=0.005), had a tumour size greater than 5 cm (P=0.018) and were mostly sarcomatous (P=0.018) (Table 4).

**Table 4:** CD10 staining exploration by pathologic parameters

Pathological parameters		CD10 − N (%)	CD10 + N (%)	P-value
**Age**	**<50**	5 (100%)	0 (0%)	0.005
	**≥50**	0 (0%)	3 (100%)	
**Histological type**	**Benign**	5 (100%)	0 (0%)	0.018
	**Borderline**	0 (0%)	1 (33.3%)	
	**Sarcoma**	0 (0%)	2 (66.7%)	
**Tumor size**	**≤2 cm**	2(40%)	0 (0%)	0.018
	**2-5 cm**	3(60%)	0 (0%)	
	**≥5 cm**	0(0%)	3 (100%)	

### EGFR and CD 10 simultaneous expression

Coexistence of CD10 and EGFR overexpression was found in 2 out of 8 patients (25%) and was associated with age, tumor size and with the aggressive histologic type of phyllodes tumors (sarcoma), data are shown in Table 5, in contrast, in the group of tumors underestimating CD10 and EGFR proteins, patients had benign phyllodes tumors (100%).

**Table 5:** Coexistence of CD 10 and EGFR in correlation with pathologic parameters

Pathologic parameters		CD10- /EGFR- n=5	CD10+/EGFR+ n=2	P-value
Age	<50	5 (100%)	0 (0%)	0.008
	≥50	0 (0%)	2 (100%)	
Tumor size	≤2 cm	02 (40%)	00 (0%)	0.030
	2-5 cm	03 (60%)	00 (0%)	
	≥5cm	00 (0%)	02 (100%)	
Histologic types	Benign	05 (100%)	0 (0%)	0.014
	Borderline	00 (0%)	0 (0%)	
	Sarcoma	00 (0%)	2 (100%)	

### PCA analysis

**Figure 4:**
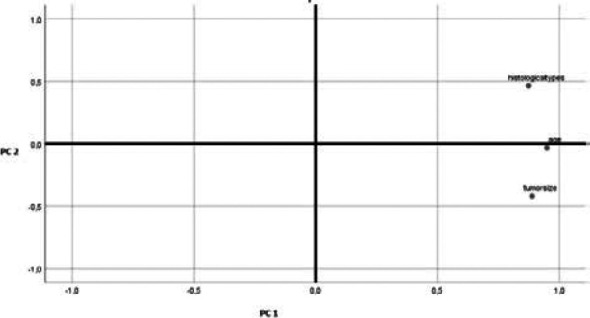
Projection of 3 variables on the plane of PC1 and PC2 from principal component analysis (PCA)

**Figure 5:**
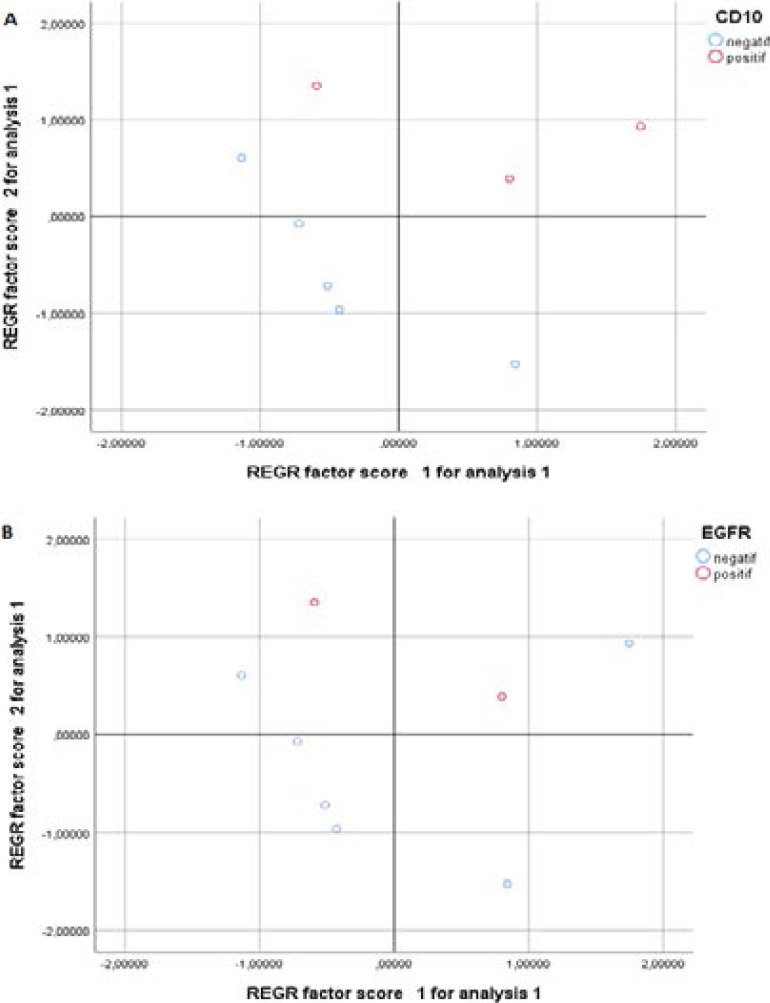
Projection of 8 points denoting patients in the space (described by 3 variables Age, histological types and tumor size) in the space of the first two principal components, PC1 and PC2, from principal component analysis (PCA). A) Projection of 8 points denoting patients expressing or not CD10 protein. B) Projection of 8 points denoting patients expressing or not EGFR protein

Principal components analysis (PCA, for short) is a variable-reduction technique that shares many similarities to exploratory factor analysis. Its aim is to reduce a larger set of variables into a smaller set of ‘artificial’ variables, called ‘principal components’, which account for most of the variance in the original variables. In this study PCA test was used to identify correlations between data points. Two principal components, PC1 and PC2, were extracted from the input data after verification of the fulfilment of the conditions for the application of this statistic test. PC1 and PC2 accounted 81.7% and 13% of the variance of the data analysed, respectively. Age and histological types are variables considered associated with bad prognosis, except tumor size located in the lower side of Figure 4. The projection of points assigned to individual patients (principal component “scores”) in the space determined by the first two principal components axes, PC1 and PC2, is depicted in Figure 5. Lower values of PC2 for patients (blue circles in Figure 5) suggest lack of CD10 and EGFR expression among patients. On the basis of PCA analysis it could be assumed that patient's age and histological type of tumor have significant association with CD10 and EGFR expression in phyllode breast tumors (Figure 4).

## Discussion

It is important to classify phyllodes tumors according to grades for predicting the recurrence and prognosis. However, to day, there is a difficulty in distinguishing breast phyllodes tumors especially borderline and sarcomas ones, inducing an underestimation of the tumour's grade and therefore the administration of an inappropriate treatment to patients.

Multiple immunohistochemistry markers have undergone a study in an attempt to improve the classification of PT and predict their outcomes. Studies demonstrate that p53, Ki67, CD117, EGFR, p16, and VEGF (being the lowest in benign phyllodes tumors and the highest in malignant phyllodes tumors) are associated with histologic grades of phyllodes tumors, but none has been proven to be clinically useful^11^,^12^,^13^,^14^.

CD10 may be a useful adjuvant in assessing the fibroepithelial neoplasms of the breast. Indeed, there have been studies that maintain that CD 10 is progressively overexpressed from fibroadenoma to sarcoma-type breast phyllodes tumors^15^. However, other authors questioned the effectiveness of stromal CD10 immunostaining for tumor grading because of the low sensitivity and suggested that combination of CD10 and smooth muscle actin or other factors will provide more information about malignant potential of phyllodes tumors^16^.

In breast cancer, EGFR seems to be involved in the pathogenesis and progression. However, still, the evaluation of EGFR as a potential biomarker in phyllodes tumors has not been well established, moreover, Tse^12^ suggested that the activation of EGFR mediated by EGF-family ligands is not the only mechanism by which proliferation occurs in phyllodes tumors. In a large study analysing 453 phyllodes tumors, the expression of EGFR in phyllodes tumors indicated by immunostaining, progressively increased from benign to malignant tumors^12^. In other study, EGFR over-expression was detected in 12.5% of benign, in 10% of borderline and 63% of all malignant phyllodes tumors. There are significant correlations between tumor grade on the one hand and EGFR overexpression^17^.

As we stated before; biomarkers defining malignancy degree still not universally established, and studies' results remain sometimes controversial, therefore, in the present study, we analysed CD10 and EGFR proteins expression, correlating them with some biopathologic parameters with the aim of obtaining insight on the behave of these two proteins on the tumor especially when they are both overexpressed in the same patient's tumor.

The results reported here, demonstrate that both EGFR and CD10 are poor prognosis factors since their overexpression were associated with a large tumour size, also we were able to point out that EGFR was overexpressed in patients with a high mitotic index (Ki67>50%). The results indicate also, as in the litterature^15^,^18^, that CD10 and EGFR are related with the degree of malignancy, in fact, CD 10 was found to be twice overexpressed in sarcomas than in borderline phyllodes tumors, while EGFR was exclusively overexpressed in sarcoma type phyllodes tumors, in addition, it has been showed that the expression of CD10 can be used to predict the occurrence of distant metastasis in phyllodes tumors of the breast^19^.

More interesting, our findings suggest the aggressive potential of tumors behaviour with simultaneous CD10 and EGFR expression since tumors were malignant, CD 10 and EGFR co expression was noted in head and neck squamous cell carcinoma^20^ and in basal breast cell lines^21^, our results may indicate that multiple signalling pathways are simultaneously active in this phyllode tumor type, in fact, amplified EGFR interplay with caveolin-1, eps15, pAkt, mdm2 and pERK and seems to present a major molecular pathway in carcinogenesis and progression of breast phyllodes tumours^22^ . overexpression of EGFR protein (with or without EGFR gene amplification or mutations) in BPTs with other upregulated genes was also found in other study^23^, which could lead to the use anti-EGFR (e.g., erlotinib/gefitinib/cetuximab) therapy^24^.

The prognostic factors identified by PCA as Bloom grade and tumour size are well known in breast cancer prediction^25^,^26^. The undertaken PCA analysis recognized that age and histological types are associated with expression status of CD 10 and EGFR in breast phyllode tumors. The limitation of our study is that we could not correlate our results with clinical outcome and follow up of the patients since our work was a single center study, also, the sample size is limited due to the rarity of BPTs in this center.

## Conclusion

This study provides additional support for comprehensive profiling in BPTs. The presented results suggest that EGFR and CD10 overexpressed combined proteins in the phyllode tumor constitute, with histopathological parameters, an important prognostic factor.
